# Phytohormone-mediated interkingdom signaling shapes the outcome of rice-*Xanthomonas oryzae* pv. *oryzae* interactions

**DOI:** 10.1186/s12870-014-0411-3

**Published:** 2015-01-21

**Authors:** Jing Xu, Lian Zhou, Vittorio Venturi, Ya-Wen He, Mikiko Kojima, Hitoshi Sakakibari, Monica Höfte, David De Vleesschauwer

**Affiliations:** Lab of Phytopathology, Department of Crop Protection, Ghent University, Coupure Links 653, 9000 Ghent, Belgium; State Key Laboratory of Microbial Metabolism, School of Life Sciences and Biotechnology, Shanghai Jiao Tong University, Shanghai, 200240 China; International Centre for Genetic Engineering and Biotechnology, Padriciano 99, 34149 Trieste, Italy; RIKEN Center for Sustainable Resource Science, Yokohama, 230-0045 Japan

**Keywords:** Plant immunity, *Oryza sativa*, Defense, Hormone signaling, Quorum sensing, Pathogenicity

## Abstract

**Background:**

Small-molecule hormones are well known to play key roles in the plant immune signaling network that is activated upon pathogen perception. In contrast, little is known about whether phytohormones also directly influence microbial virulence, similar to what has been reported in animal systems.

**Results:**

In this paper, we tested the hypothesis that hormones fulfill dual roles in plant-microbe interactions by orchestrating host immune responses, on the one hand, and modulating microbial virulence traits, on the other. Employing the rice-*Xanthomonas oryzae* pv. *oryzae* (*Xoo*) interaction as a model system, we show that *Xoo* uses the classic immune hormone salicylic acid (SA) as a trigger to activate its virulence-associated quorum sensing (QS) machinery. Despite repressing swimming motility, sodium salicylate (NaSA) induced production of the Diffusible Signal Factor (DSF) and Diffusible Factor (DF) QS signals, with resultant accumulation of xanthomonadin and extracellular polysaccharides. In contrast, abscisic acid (ABA), which favors infection by *Xoo*, had little impact on DF- and DSF-mediated QS, but promoted bacterial swimming via the LuxR solo protein OryR. Moreover, we found both DF and DSF to influence SA- and ABA-responsive gene expression *in planta*.

**Conclusions:**

Together our findings indicate that the rice SA and ABA signaling pathways cross-communicate with the *Xoo* DF and DSF QS systems and underscore the importance of bidirectional interkingdom signaling in molding plant-microbe interactions.

**Electronic supplementary material:**

The online version of this article (doi:10.1186/s12870-014-0411-3) contains supplementary material, which is available to authorized users.

## Background

Bacterial leaf blight (BLB) is one of the most devastating diseases on rice, causing annual yield losses up to 60% [1]. The causal agent, *Xanthomonas oryzae* pv. *oryzae* (*Xoo*) is a rod-shaped, obligately aerobic, gram-negative bacterium that is motile by means of a single polar flagellum. Like many other *Xanthomonas* species, *Xoo* produces a wide variety of virulence factors to protect itself and inflict disease. These factors include extracellular polysaccharides (EPS), lipopolysaccharides, adhesins, cell wall degrading enzymes, and type III effectors [2,3]. Most of these traits are under tight control of quorum sensing (QS) regulatory systems.

QS is a cell-to-cell communication system by which bacteria track changes in cell density and adjust gene expression accordingly. Central to QS is the production, detection, and response to extracellular signal molecules called autoinducers (AIs). At low cell density, bacteria produce basal levels of AIs, which subsequently diffuse away in the environment, preventing detection by bacterial receptor proteins. However, as the population density reaches a certain threshold level, accumulated AIs are easily detected, setting off a variety of biological processes including EPS biosynthesis, motility, competence, and virulence factor secretion [4-6]. As such, QS enables unicellular bacteria to act as multicellular organisms and invest in energy-consuming processes only when the impact of these processes on the environment or on a host will be maximized.

It is well-established that *Xoo* produces different types of AIs, including the Diffusible Signal Factor (DSF) molecule *cis*-11-methyl-2-dodecenoic acid [7-11]. Originally identified in *Xanthomonas campestris* pv. *campestris* (*Xcc*), DSF and its derivatives represent a relatively novel family of QS signals that is widely conserved among Gram-negative bacterial species. So far, different DSF-family signals have been identified and functionally characterized in a range of plant and human bacterial pathogens, including *Xanthomonas campestris* pv. *campestris* (*Xcc*), *Xyllela fastidiosa*, *Stenotrophomonas maltophilia*, and *Burkholderia cenocepacia* [7,8,12-14]. Biosynthesis of DSF depends on *rpfB* and *rpfF*, which encode a fatty acyl-CoA ligase and a putative enoyl-coA hydratase, respectively [7,11]. According to recent findings, RpfB is not directly involved in DSF biosynthesis but influences fatty acid recycling to counteract the thioesterase activity of RpfF [15]. Downstream of DSF synthesis, perception and transduction of DSF signals occurs through a conserved phosphorelay mechanism governed by the RpfC/RpfG two-component system [16-18]. Interestingly, RpfC not only senses DSF, but also negatively regulates DSF biosynthesis via direct protein-protein interactions [19], while RpfG transmits DSF signals by influencing the level of the secondary QS messenger cyclic-di-GMP. Consistent with DSF signaling playing a vital role in *Xoo* virulence, null mutants of *rpf*C and *rpf*G display reduced xylanase activity, swimming ability and EPS synthesis, and consequently, cause little disease when inoculated on rice plants [9].

In addition to DSF-governed QS, there is evidence for a second QS circuit in *Xoo* that appears to be mediated by a Diffusible Factor (DF) characterized as 3-hydroxybenzoic acid (3-HBA). As in *Xcc*, this DF system has been found to regulate the production of yellow, membrane-bound pigments, known as xanthomonadins [20]. Of particular note, DF not only controls the biosynthesis of xanthomonadins but also is incorporated into the metabolite itself, questioning its status as a QS signal *sensu stricto*. Besides being a diagnostic characteristic of the genus [21,22], xanthomonadins play essential roles in protecting bacteria from photobiological and peroxidative damage and are involved in epiphytic survival and systemic plant infection [21,23,24]. Goel et al. (2002) demonstrated that xanthomonadin synthesis in *Xoo* strain BXO1 is encoded by a 21 kB gene cluster that contains seven transcriptional units, designated *pigA* to *pigG* [25]. Analysis of the *pigB* DNA sequence revealed the presence of two open reading frames, the second one (*xanB2*) being responsible for synthesis of DF [26,27]. Recent results indicate that XanB2 also hydrolyzes chorismate to produce 4-HBA, which is mainly used as a precursor for coenzyme Q (CoQ) biosynthesis [20].

In addition to the canonical DSF- and DF-mediated QS systems that consist of a LuxI-family synthase responsible for synthesizing the QS signal and a cognate LuxR-family transcriptional regulator that binds QS factors at quorum concentrations, *Xoo* also harbors the LuxR solo protein OryR. Lacking of cognate LuxI proteins, LuxR solos regulate target genes by either sensing endogenous QS factors or by ‘eavesdropping’ on exogenous factors produced by neighbouring bacteria. However, some solos can also respond to low-molecular weight compounds produced by plants. OryR, for instance, is known to interact with an unknown rice signal molecule (RSM) to activate plant virulence genes [28]. As such, LuxR-like solos function as messengers of both interspecies and interkingdom signaling [29].

In plants, signaling pathways are typically controlled by small-molecule hormones. Upon pathogen attack, plants synthesize a complex blend of hormones. This so-called hormone signature is well known to play a key role in the orchestration of plant immune responses and to determine the specific nature of the defense mechanism triggered [30,31]. However, exciting new developments suggest that plant hormones not only steer plant immune responses, but also may influence the pathogen’s virulence machinery. For instance, a recent study showed that SA reduces virulence of *Agrobacterium tumefaciens* by inhibiting the virA/G two-component system [32]. Moreover, in the opportunistic human pathogen *Pseudomonas aeruginosa*, SA was reported to reduce the production of several virulence factors including motility, biofilm formation and AI production [33-35].

In view of these findings, we hypothesized that plant hormones may fulfill a dual role in plant-bacteria interactions, modulating plant immune responses, on the one hand, and acting as (ant)agonists of bacterial QS systems on the other. To test this hypothesis, we investigated the effect of the plant hormones abscisic acid (ABA) and salicylic acid (SA) on several QS-regulated virulence traits of *Xoo*, including motility, production of EPS and xanthomonadin synthesis. SA is a positive regulator of resistance to *Xoo* [36], whereas ABA antagonizes SA and acts as a susceptibility-enhancing hormone that is usurped by *Xoo* in order to cause disease [37]. Reversely, we also tested the effect of the *Xoo* QS signals DSF and DF on the rice SA and ABA pathways. Together, our findings favor a scenario whereby SA and ABA cross-communicate with the DFS and DF QS circuits to modulate *Xoo* virulence and underscore the importance of bidirectional interkingdom signaling in molding plant-bacteria interactions.

## Results

### NaSA suppresses bacterial swimming in a dose-dependent manner

**Figure 1:**
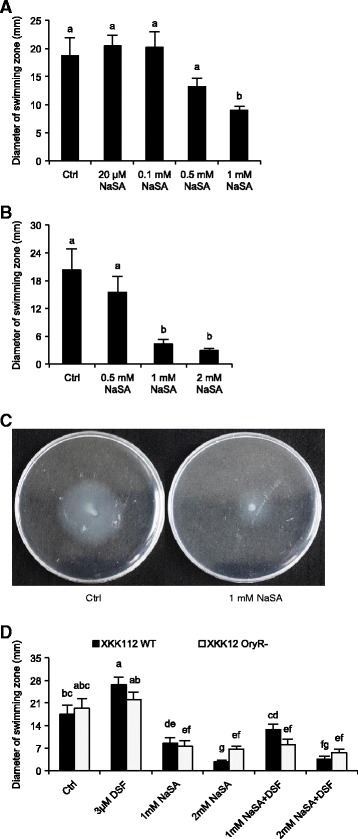
**NaSA suppresses basal and DSF-induced swimming motility of** ***Xanthomonas oryzae*** **pv.** ***oryzae*** **(** ***Xoo*** **) strain XKK12 (pPIP122). (A)** and **(B)** Various concentrations of NaSA were added to swimming plates prior to inoculation with 3 μl XKK12 WT (pPIP122) suspension (10^9^ CFU/ml). The plates were incubated for four days at 25°C and evaluated by measuring the diameter of the swimming zone. Data are means ± SE. Different letters indicate statistically significant differences (Mann-Whitney: n ≥ 9; α = 0.05). **(C)**, Phenotype of XKK12 WT (pPIP122) on swimming plates containing 0 (left) or 1 mM NaSA (right). **(D)**, Effects of 1 mM NaSA, 2 mM NaSA and/or 3 μM DSF on the swimming of XKK12 WT (pPIP122) and *oryR* knockout mutant (pPIP122) (*oryR* ^*−*^) bacteria. Data are means ± SE. Different letters indicate statistically significant differences (Mann-Whitney: n ≥ 9; α = 0.05).

Mounting evidence indicates that SA is an important player in the activation of rice defenses against *Xoo* [36,38]. In a first attempt to test whether SA also exerts direct effects on the QS machinery of *Xoo*, we evaluated the effect of SA on the swimming ability of *Xoo* strain XKK12 (pPIP122). Unless specified otherwise, this strain was routinely used throughout this paper. Since SA showed strong inhibition of bacterial growth in both liquid and solid medium in our preliminary trials, all following experiments were performed using sodium salicylate (NaSA), which had no significant impact on XKK12 growth rates on (semi-)solid agar plates (data not shown). Swim plates indicated that XKK12 is motile in swimming medium, reaching a diameter of about 20 mm after 4-6 days of incubation at 25°C. As shown in Figures 1A to 1C, NaSA at concentrations of 20 μM, 0.1 mM and 0.5 mM had little or no significant effect on the swimming ability of XKK12, whereas 1 mM and 2 mM of NaSA significantly reduced swimming. SA concentrations in basal rice leaves range from 5 to 20 μg/g fresh weight [39]. To shed light on the SA concentrations in pathogen-inoculated rice leaves, we used a previously published UPLC-MS/MS method to profile the hormone signature of *Xoo*-infected rice plants at various times post inoculation [40]. Consistent with previous reports, SA levels remained fairly constant throughout the course of infection, except for a late two-fold increase at 8 dpi (Additional file 1). At this time point, SA levels reached 613235 ± 12043 pmol/g FW, which corresponds to 0.767 mM SA. Supplying bacteria with 1 or 2 mM NaSA thus resembles the amount of SA encountered by *Xoo in planta*, demonstrating the physiological relevance of these concentrations.

Motility of *Xoo* is under control of the DSF QS system, with DSF signaling either promoting or suppressing swimming depending on the bacterial strain [9,41,42]. Under our experimental conditions, 3 μM DSF, which is the lowest concentration reported to be effective in *Xoo* [42], significantly increased swimming of XKK12 (Figure 1D). Interestingly, co-application of 3 μM DSF and 1 mM NaSA alleviated the swimming-promoting effect of single DSF treatments, while 2 mM NaSA completely abolished it (Figure 1D). Together these results suggest that NaSA suppresses swimming of XKK12 in a concentration-dependent manner, possibly by antagonizing DSF-mediated signaling.

Swimming motility of *Xoo* was previously shown to be controlled by OryR, which belongs to a sub-family of the *N*-acyl homoserine lactone (AHL) responding LuxR regulators [28,43]. To assess whether NaSA suppresses swimming in an OryR-dependent manner, we tested the effect of NaSA on WT bacteria and the *oryR* knockout mutant (*oryR*^*−*^). Consistent with earlier findings by Gonzalez et al. [43] demonstrating that the effect of OryR on bacterial swimming is only observed upon addition of macerated rice extract to the medium, WT and *oryR*^*−*^ mutant bacteria swam to a similar extent when grown on control plates (Figure 1D). Perhaps more surprisingly, the oryR^−^ mutant proved to be non-responsive to DSF-induced swimming, while NaSA treatment turned out to be equally effective in the WT and mutant background (Figure 1D). Thus, OryR seems to be indispensable for DSF-induced swimming, whereas NaSA may suppress swimming independently of OryR.

### NaSA induces EPS production in XKK12 via OryR

**Figure 2:**
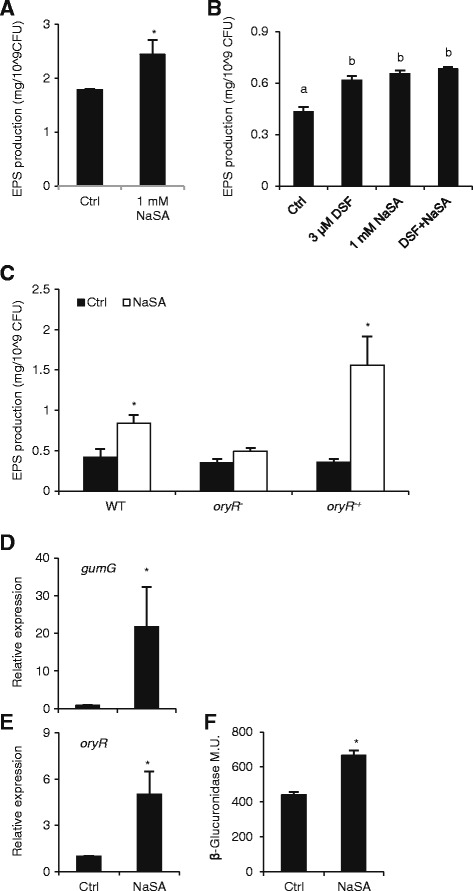
**NaSA stimulates EPS synthesis in XKK12 WT (pPIP122) in an OryR-dependent manner. (A)**, EPS production of XKK12 WT (pPIP122) grown on PSA plates with or without 1 mM NaSA. Data are means ± SE of three plates. Asterisks indicate statistically significant differences compared to the control (T-test: n = 3; α = 0.05). **(B)**, Effects of 1 mM NaSA and/or 3 μM DSF on EPS production of XKK12 WT (pPIP122) grown in PY broth. Data are means ± SE of three replicates. Different letters indicate statistically significant differences (Tukey: n = 3; α = 0.05). **(C)**, Effect of 1 mM NaSA on EPS production of XKK12 WT, *oryR* ^*−*^ and *oryR* ^*−*^ complemented strain (*oryR* ^*-+*^) grown in PY broth. Data are means ± SE of three independent experiments. Asterisks indicate statistically significant differences compared to the control (T-test: n = 3; α = 0.05). **(D)** and **(E)**, Effect of 1 mM NaSA on expression profiles of *oryR* and the EPS biosynthesis gene *gumG* in PY-grown XKK12 WT (pPIP122). Data are means ± SE of two technical and two biological replicates. Asterisks indicate statistically significant differences compared to the control (T-test: n = 4; α = 0.05). **(F)**, *oryR* gene promoter activity in XKK12 WT harboring the reporter plasmid pORYR122 (Ferluga and Venturi, 2009) grown in PY broth supplemented or not with 1 mM NaSA. Data are means ± SE of three replicates from a representative experiment. The experiment was repeated twice with similar results. Asterisks indicate statistically significant differences compared to the control (T-test: n = 3; α = 0.05).

In many bacteria, synthesis of extracellular polysaccharides (EPS) is a key determinant of biofilm formation and, hence, an inhibitory factor of motility [44,45]. Given that NaSA inhibits swimming of XKK12, we sought to extend our analysis by testing the influence of NaSA on biofilm formation and EPS synthesis of *Xoo*. Unfortunately, classic biofilm assays using Crystal Violet staining failed to show consistent bacterial attachment to polystyrene culture plates, suggesting that XKK12 does not form biofilms under the conditions we tested (data not shown). When XKK12 was grown on solid peptone sucrose agar (PSA) plates, NaSA showed no effect on bacterial growth but significantly enhanced EPS synthesis (Figure 2A). In several preliminary assays liquid XKK12 cultures grown in the presence of 1 mM NaSA also consistently produced more EPS than the control treatment at 24 h post inoculation (OD control: 3.15 ± 0.53, OD NaSA treatment: 2.78 ± 0.53, EPS production control: 0.40 ± 0.07 mg/OD, EPS production NaSA treatment: 0.75 ± 0.09 mg/OD – data from 5 different experiments). Therefore, and given the tractability of liquid assays, all follow-up experiments were performed using liquid XKK12 cultures.

Previous work revealed that EPS production is impaired in the *Xoo rpf*F knockout mutant but restored by exogenous addition of DSF [9,41,42,46], suggesting a positive role for DSF in regulating EPS production in *Xoo*. Consistent with these results and as shown in Figure 2B, 3 μM DSF significantly increased EPS synthesis in XKK12. A similar promoting effect was observed for 1 mM NaSA, whereas co-application of both compounds had no additive effect. Interestingly, NaSA-induced EPS synthesis was much less pronounced in the *oryR*^*−*^ mutant background but restored in the *oryR*^*−*^ complemented strain (Figure 2C), suggesting that NaSA stimulates EPS production in XKK12 in an OryR-dependent manner. Consistent with this hypothesis, qRT-PCR analysis showed that 1 mM NaSA strongly up-regulated the expression of both *oryR* and the EPS biosynthesis gene *gumG* compared with control treatments (Figures 2D and 2E). Moreover, β-glucuronidase reporter assays revealed that NaSA also caused an approximate 50% increase in *oryR* promoter activity (Figure 2F).

### NaSA stimulates DSF signaling pathway by inducing DSF biosynthesis

**Figure 3:**
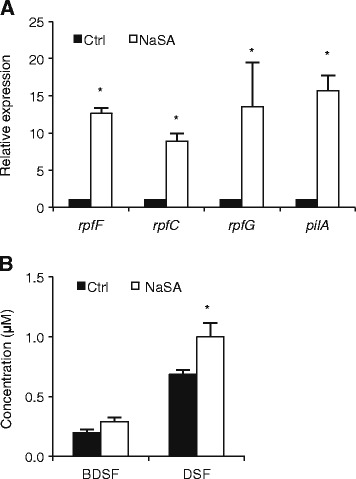
**Effect of NaSA on DSF biosynthesis and signaling. (A)**, Expression of DSF biosynthesis and responsive genes *rpfF*, *rpfC*, *rpfG*, and adhesin-encoding *pilA* in XKK12 WT (pPIP122) grown in PY broth supplemented or not with 1 mM NaSA. Data are means ± SE of two technical and two biological replicates. Asterisks indicate statistically significant differences compared to the control (T-test: n = 4; α = 0.05). **(B)**, Quantification of DSF and BDSF produced by XKK12 WT (pPIP122) grown in PY broth containing 0 or 1 mM NaSA. Data are means ± SE of three independent experiments. Asterisks indicate statistically significant differences compared to the control (T-test: n = 3; α = 0.05).

The finding that NaSA induces transcription of the LuxR solo *oryR* led us to analyze whether NaSA likewise influences the DSF QS system. For this purpose, we first tested the effect of NaSA on the expression of various genes located in the DSF signaling cascade. *rpfF*, *rpfC*, and *rpfG* are DSF biosynthesis, response and signaling genes, respectively [9,47], while *pilA* encodes an adhesion protein involved in biofilm formation and motility [9]. Remarkably, all of these genes were several fold up-regulated by 1 mM NaSA treatment, suggesting that NaSA activates the entire DSF pathway, possibly by inducing DSF biosynthesis. To test this hypothesis, we quantified the effect of NaSA on the biosynthesis of DSF and the DSF-like signals BDSF and CDSF using HPLC [42]. Since DSF levels in wild-type *Xoo* are too low to be reliably measured, an DSF-overproducing *rpfC* mutant strain was used in these assays [42]. As shown in Figure 3B, NaSA did not significantly influence BDSF production but caused a nearly 50% increase in DSF levels, while CDSF remained below the quantification limit.

### NaSA induces 3-HBA and 4-HBA production in XKK12

**Figure 4:**
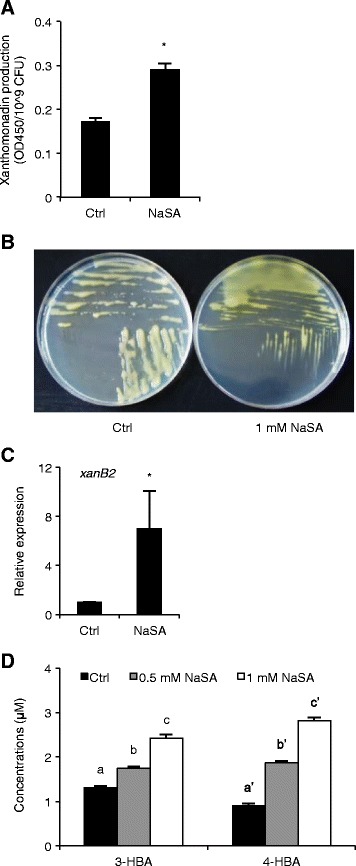
**NaSA induces the DF QS circuit of** ***Xoo*** **with resultant accumulation of xanthomonadin. (A**), Effect of 1 mM NaSA on xanthomonadin production of XKK12 WT (pPIP122) grown in PY broth. Data are means ± SE of three plates. Asterisks indicate statistically significant differences compared to the control (T-test: n = 3; α = 0.05). **(B)**, Yellow pigmentation of XKK12 WT (pPIP122) grown on PSA plates containing 0 (left) or 1 mM NaSA (right). **(C)**, Expression of xanthomonadin and DF biosynthesis gene *xanB2* in XKK12 WT (pPIP122) grown in PY broth containing 0 or 1 mM NaSA. Data are means ± SE of two technical and two biological experiments. Asterisks indicate statistically significant differences compared to the control (T-test: n = 4; α = 0.05). **(D)**, Quantification of 3-HBA (DF) and 4-HBA produced by XKK12 WT (pPIP122) grown in PY broth supplemented or not with 0.5 or 1 mM NaSA. Data are means ± SE of two technical and two biological replicates. Different letters indicate statistically significant differences (Tukey: n = 4; α = 0.05).

In addition to OryR and the DSF QS system, *Xoo* is equipped with an alternative QS circuit that is mediated by the DF signal and controls production of xanthomonadin. Interestingly, XKK12 bacteria grown on PSA plates containing 1 mM NaSA produced about 60% more xanthomonadin compared with the control (Figures 4A and 4B). Consistent with these findings, NaSA-supplemented bacteria also displayed increased expression of the xanthomonadin- and DF-biosynthesis gene *xanB2* (Figure 4C). Moreover, HPLC-based measurements revealed that NaSA stimulates the production of 3-HBA (DF) and 4-HBA in a dose-dependent manner (Figure 4D). Besides inducing DSF signaling, NaSA thus also appears to activate the DF QS circuit, with resultant accumulation of xanthomonadin.

### ABA promotes swimming of XKK12 in an OryR-dependent manner

**Figure 5:**
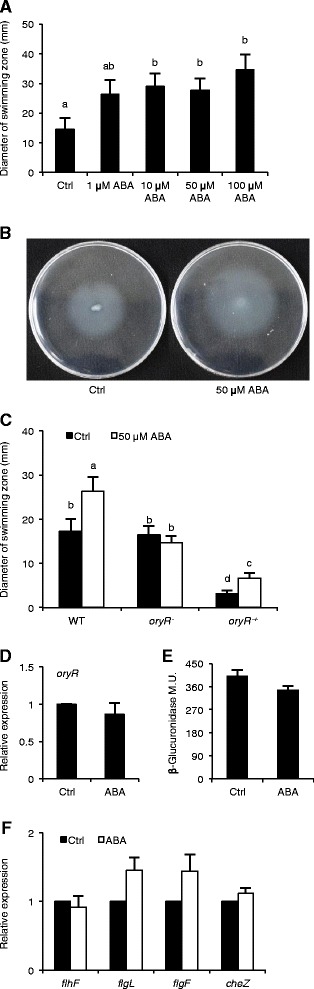
**Abscisic acid (ABA) promotes swimming of XKK12 WT (pPIP122) via the LuxR solo OryR. (A)**, Effect of different ABA concentrations on swimming motility of XKK12 WT (pPIP122). Equivalent volumes of the ABA solvent ethanol (EtOH) were added to control (Ctrl) treatments. Data are means ± SE. Different letters indicate statistically significant differences (LSD: n ≥ 9; α = 0.05). **(B)**, Phenotype of XKK12 WT (pPIP122) on swimming plates containing 0 (left) or 50 μM ABA (right). **(C)**, Effect of 50 μM ABA on the swimming of XKK12 WT, *oryR* ^*−*^ and the complemented stain *oryR* ^*-+*^. Data are means ± SE of three independent experiments. Different letters indicate statistically significant differences (Mann-Whitney: n ≥ 18; α = 0.05). **(D)**, Expression of *oryR* in XKK12 WT (pPIP122) grown in PY broth with or without 50 μM ABA. Data are means ± SE of two technical replicates and two biological replicates. There were no significant differences between control (Ctrl) and ABA treatments. (T-test: n = 4; α = 0.05). **(E)**, *oryR* gene promoter activity in XKK12 WT harboring the pORYR122 reporter plasmid and grown in PY broth supplemented or not with 50 μM ABA. Data are means ± SE of three replicates from a representative experiment. There were no significant differences between control (Ctrl) and ABA treatments (T-test: n = 3; α = 0.05). Repetition of experiments led to results very similar to those shown. **(F)**, Expression of OryR-regulated flagellar genes in XKK12 WT (pPIP122) grown in PY broth in response to 50 μM ABA. Data are means ± SE of two technical and two biological replicates. There were no significant differences between control (Ctrl) and ABA treatments. (T-test: n = 4; α = 0.05).

Previously, we showed that ABA antagonizes SA signaling in rice, thereby favoring BLB disease development [37]. In a first attempt to investigate whether ABA, like SA, acts as a QS (ant)agonist, we tested the effect of increasing concentrations of ABA on the swimming ability of XKK12. After 4 days of incubation, bacteria cultured on plates containing either 10 μM, 50 μM or 100 μM of ABA showed enhanced swimming compared to the solvent-treated controls, whereas 1 μM ABA had no significant effect (Figures 5A and 5B). Importantly, none of these concentrations had a significant impact on the growth characteristics of XKK12 (data not shown), suggesting that ABA concentrations above 1μM promote swimming without any clear dose-response.

Given the positive role of OryR in promoting bacterial swimming, we next compared the effect of 50 μM ABA on WT and *oryR*^*−*^ bacteria. Interestingly, ABA treatment was ineffective in the *oryR*^*−*^ background whereas it significantly enlarged the swimming zone in WT bacteria (Figure 5C). Consistent with previous findings, complementing the *oryR*^*−*^ mutant by introducing the *oryR* gene in the high-copy plasmid pBBROryR significantly decreased swimming as compared to WT bacteria, a phenomenon which is likely explained by the overexpression of *oryR* in a multicopy plasmid and the self-negative regulation of *oryR* [43]. Nevertheless, complementation of *oryR-* did restore ABA-induced swimming, indicating that OryR is indispensable for ABA-mediated swimming (Figure 5C).

To test whether ABA, like SA, affects *oryR* expression, we next investigated the transcriptional behavior of *oryR* in XKK12 WT pPIP122 grown in PY broth containing 50 μM ABA. As shown in Figure 5D, ABA had little effect on the expression of *oryR* and, according to our β-glucuronidase assays, also failed to induce *oryR* promoter activity (Figure 5E). Moreover, growing bacteria in the presence of ABA did not significantly alter the expression of the OryR-regulated flagellar genes *flgL*, *flgF*, *flhF* and *cheZ* [43], suggesting that ABA-induced swimming of *Xoo* does not involve transcriptional activation of *oryR* or its target genes (Figure 5F).

### ABA has little impact on the DSF and DF QS circuits

**Figure 6:**
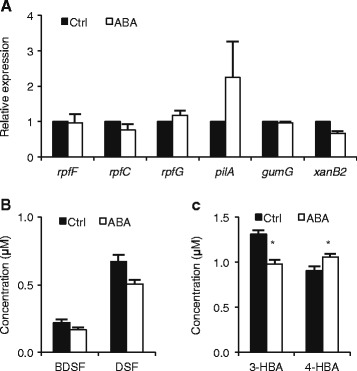
**ABA has little impact on the DSF- and DF-type QS circuits in** ***Xoo*** **. (A)**, Expression of DSF biosynthesis and responsive genes *rpfF*, *rpfC*, and *rpfG*, EPS biosynthesis gene *gumG*, adhesion gene *pilA*, and xanthomonadin biosynthesis gene *xanB2*, in XKK12 WT (pPIP122) grown in PY broth in response to 50 μM ABA. Data are means ± SE of two technical and two biological replicates. There were no significant differences between control (Ctrl) and ABA treatments. (T-test: n = 4; α = 0.05). **(B)** and **(C)**, Quantification of DSF and DF produced by XKK12 WT (pPIP122) grown in PY broth with or without 50 μM ABA. Data are means ± SE of three independent experiments. Asterisks indicate statistically significant differences compared to the control (T-test: n = 4; α = 0.05).

The finding that NaSA induces several genes located in the DSF and DF QS pathways (Figures 2, 3 and 4) prompted us to check whether ABA exerted a similar effect. However, as shown in Figure 6A, ABA had no statistically significant impact on the expression of any of the DF and DSF-related genes tested. ABA also failed to significantly alter the synthesis of BDSF and DSF, but slightly repressed 3-HBA and weakly enhanced 4-HBA synthesis, which has also been implicated in EPS and xanthomonadin regulation [48] (Figures 6B and 6C). Nevertheless, we found ABA to be ineffective in both EPS and xanthomonadin assays (data not shown). Except for stimulating swimming via a yet to be defined mechanism, ABA therefore seems to have little effect on the DSF and DF QS circuits, neither on the virulence traits mediated by them.

### DSF and DF differentially impact rice ABA and SA signaling pathways

**Figure 7:**
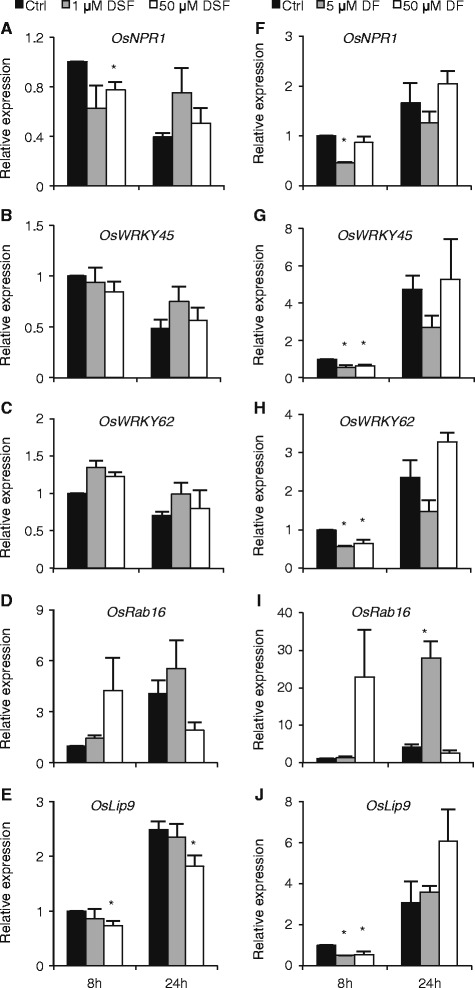
**Effects of DSF and DF on ABA and SA signaling pathways in rice.** 6-week-old Taipei leaf segments were incubated in aqueous solutions containing different concentrations of DSF **(A to E)**, DF **(F to J)**, or equivalent volumes of solvent (Ctrl), and sampled at 8 and 24 hours post treatment (hpt). Expression of the ABA responsive genes *OsLip9* and *OsRab16* and the SA marker genes *OsWRKY45*, *OsNPR1* and *OsWRKY62*, was determined by qRT-PCR. Data are means ± SE of two technical and two biological replicates. Asterisks indicate statistically significant differences compared to the control (T-test: n = 4; α = 0.05). Repetition of experiments led to results very similar to those shown.

Apart from coordinating expression of microbial virulence genes, bacteria-produced QS signals have also been shown to interfere with the host hormone signaling circuitry [49,50]. Therefore and given the importance of SA-ABA signal interactions in BLB resistance [37], we evaluated the impact of exogenously administered DSF and DF on rice SA and ABA signaling pathways. To this end, detached leaves of 6-week-old rice Taipei plants were treated with either low or high concentrations of DSF and DF and tested for expression of several ABA and SA-responsive marker genes. As shown in Figure 7, expression of the SA marker genes, *OsWRKY45*, *OsWRKY62* and *OsNPR1* was only weakly responsive to either 1 or 50 μM DSF, suggesting that DSF has little, if any, impact on the rice SA pathway. In addition, 50 μM DSF barely altered the expression of the ABA marker gene *OsRab16* at either 8 or 24 hours post treatment while it had a significant albeit minor suppressive effect on transcription of another marker gene *OsLip9* (hpt; Figures 7D and 7E). Suppression of *OsLip9* was also observed in response to DF at 8 hpt, whereas *OsRab16* was upregulated by 5 μM DF at 24 hpt (Figures 7I and 7J). In addition, both 5 μM and 50 μM DF treatments significantly lowered transcription of *OsNPR1*, *OsWRKY45* and *OsWRKY62* at 8 but not at 24 hpt, suggesting that DF is able to transiently suppress SA signaling.

## Discussion

The interaction between pathogens and their host plants has been the subject of intense research. Contrary to the large body of evidence demonstrating the pivotal role of hormones in orchestrating the plant immune signaling network, surprisingly little is known about whether hormones also impinge on the virulence machinery of plant pathogens, similar to what has been reported in animal systems. In one of the most notable examples, the enterohemorrhagic *Escherichia coli* (EHEC) serotype O157:H7, a pathogen responsible for outbreaks of bloody diarrhea in several countries, was shown to exploit the mammalian hormone epinephrine in order to activate its QS machinery [51]. Similarly, several reports in the literature have revealed a negative impact of the plant hormone SA on virulence gene expression in the opportunistic human pathogen *Pseudomonas aeruginosa* [33,34]. In turn, several AHL QS factors have been shown to exert immunomodulatory activities in animal hosts [52]. These studies suggest that prokaryotic-eukaryotic communication occurs through bacterial QS factors and host hormones. Here we provide the first report of a similar phenomenon with respect to the plant pathogen *Xanthomonas oryzae* pv. *oryzae* (*Xoo*), one of the most important threats of cultivated rice worldwide. Our findings favor a scenario wherein SA and ABA not only steer rice immune responses, but also cross-communicate with bacterial QS systems to regulate virulence traits in *Xoo*. Moreover, our results also imply reciprocal crosstalk with *Xoo* QS signals modulating rice SA and ABA signaling pathways. We propose that the dynamic nature and balance of such bidirectional interkingdom signaling is an important regulatory factor determining the outcome of plant-bacteria interactions.

### SA activates the *Xoo* DSF and DF QS systems to coordinately regulate swimming, EPS and xanthomonadin synthesis

Swimming and EPS production are important virulence factors of *Xoo* and both traits are considered to be under control of QS. Several lines of evidence indicate that DSF signaling positively regulates EPS production, whereas the effect of DSF on swimming of *Xoo* appears to be strain-dependent [9,41]. Under our experimental conditions, exogenously administered DSF promoted both swimming and EPS production of strain XKK12, confirming the importance of DSF signaling in these processes (Figures 1D and 2A). Consistent with previous reports in *P. aeruginosa* and *E. coli* [34,53], NaSA inhibited bacterial swimming, yet strongly promoted EPS synthesis and up-regulated various genes located both upstream (*rpf* gene cluster) and downstream (*pilA*) in the DSF signaling pathway (Figures 1, 2 and 3A). Intriguingly, we also found NaSA to significantly increase DSF production in liquid cultures (Figure 3B), providing a mechanism for how NaSA may activate the entire DSF signaling circuit and increase EPS synthesis by stimulating DSF biosynthesis.

NaSA-induced DSF synthesis may seem contradictory in light of the positive role of DSF in swimming of XKK12 and the ability of NaSA to repress the latter process. However, regulation of flagellar motility in xanthomonads is a complex multi-step process involving over 40 genes that are regulated in a hierarchical manner [54,55]. Kunin et al. [53] previously demonstrated the ability of SA to block flagella biosynthesis in *E. coli*. Therefore, it is not inconceivable that NaSA transiently activates DSF signaling, but negatively affects swimming of XKK12 by an as yet unknown mechanism, which could involve repression of individual flagella-associated genes and/or post-transcriptional modifications. In addition, it should be noted that the conditions of growth for swimming and gene expression studies are quite different, so that simple extrapolations may not be appropriate.

In addition to setting off DSF QS signaling, our data also point towards a role of NaSA in activating the DF QS system. Genetic analysis revealed that DF (3-HBA), which is synthesized by the key metabolic enzyme XanB2, catalyzes the production of xanthomonadin pigments [20,27,48]. Interestingly, xanthomonadin is synthesized from shikimate and chorismate, both of which are also important precursors for SA biosynthesis in plants [56,57]. Although the structure of SA (2-HBA) is very similar to that of DF (3-HBA), SA failed to complement the *Xcc xanB2* knockout mutant [27,23]. However, we found that NaSA enhances *xanB2* gene expression and, accordingly, triggers enhanced levels of DF and xanthomonadin (Figures 4A to 4C). Notably, DF is reported to be responsible for EPS production as well [48], suggesting that the induction of EPS by NaSA may derive at least in part from its positive effect on DF synthesis. NaSA also promoted the production of 4-HBA (Figure 4D), the second product of XanB2 [20,48]. Unlike DF, 4-HBA is responsible for the biosynthesis of CoQ, which functions as a key cofactor in the aerobic respiratory electron transfer for energy generation and protects bacteria from peroxidative damage [58]. Given the importance of DF and 4-HBA in bacterial virulence [20,48] and the well-described role of EPS and xanthomonadin in protecting bacteria from host-imposed stresses [23,24,59], one interesting extrapolation is that SA not only serves as a plant resistance-inducing signal, but also ended up being exploited by *Xoo* as a QS agonist. Moreover, in view of the idea that bacteria possess intensity ‘switches’ that ensure timely expression of energy-intensive molecular and biochemical processes [60], it is tempting to speculate that *Xoo* uses SA to ‘sense’ that is within the host vascular system and activate genes essential for plant colonization.

### The LuxR-type solo OryR coordinates crosstalk between plant SA/ABA and *Xoo* QS circuits

Belonging to a sub-family of LuxR proteins that have the same modular structure of QS LuxRs but are devoid of a cognate LuxI AHL synthase, the so-called LuxR ‘solo’ OryR controls the expression of more than 300 bacterial genes and is important for the complete virulence of *Xoo* [43,61]. LuxR solos regulate target genes by either sensing endogenous QS factors or by ‘eavesdropping’ on exogenous factors produced by neighboring bacteria [62]. However, some solos can also respond to low-molecular weight compounds produced by plants [63]. Biochemical studies have shown that OryR does not respond to a wide variety of AHL-type QS factors, but is solubilized in the presence of media supplemented with macerated rice leaves [28,61]. This indicates that OryR most probably binds a plant-produced compound, the identity of which remains unclear. Intriguingly, our data suggest that OryR is involved in the perception and/or transduction of plant hormone signals with NaSA activating both gene expression and promoter activity of *oryR* (Figure 2E and 2F). Moreover, experiments with *oryR*- knockout strains revealed that OryR is indispensable for NaSA-induced EPS synthesis and ABA-promoted swimming. However, NaSA does not solubilize OryR, suggesting that NaSA most probably does not bind OryR (data not shown). Therefore, rather than acting as a bacterial hormone receptor *per se*, OryR likely functions by integrating, processing and transmitting plant and bacterial signal molecules, thus acting as a major hub for signal integration and pathway crosstalk. Nonetheless, it should be noted that OryR is not solely determinant for bacterial hormone responses as NaSA reduced swimming to similar extents in WT and *oryR*- mutant bacteria (Figure 1D). Moreover, unlike SA, ABA treatment had no significant impact on gene expression and promoter activity of *oryR* (Figures 5D and 5E), suggesting that ABA regulates swimming of XKK12 downstream of OryR. Gene expression experiments failed to show a substantial effect of ABA on any of the OryR-dependent flagellar and chemotaxis genes we tested (Figure 5F). Since there are 17 motility-related genes that are positively regulated by OryR, it cannot be ruled out that ABA affects one or more genes that we did not check. Furthermore, ABA-mediated regulation of swimming may also occur at the post-transcriptional level. Deciphering the exact mechanism(s) by which ABA regulates OryR-dependent swimming is a key challenge for future research.

### *Xoo* QS signals (DSF and DF) modulate rice SA and ABA signaling pathways

In addition to plants producing AHL mimics that are able to act as agonists or antagonists to bacterial AHL QS systems, accumulating evidence indicates that bacterial QS factors modulate plant-microbe interactions by tapping into various plant signaling circuits [64,65]. In one of the first examples, it was reported that treatment of *Medicago truncatula* with AHLs drives transcriptional reprogramming of extensive gene sets involved in host defense responses, primary metabolism, transcriptional regulation, protein processing, cytoskeletal activity, and plant hormone responses [50]. Similarly, AHL-treated Arabidopsis displayed altered expression of selected hormone-responsive genes as well as significant changes in the plant’s hormone balance, in particular an increased auxin/cytokinin ratio [66]. In common with these findings, our data revealed that low and high concentrations of DF but not DSF transiently suppress SA-responsive gene expression in detached rice leaves. Considering the similarity in chemical structure of DF (3-HBA) and SA (2-HBA), one may envision that *Xoo*-secreted DF not only facilitates bacterial cell-to-cell communication, but also disturbs *in planta* SA homeostasis and hence disrupts host immune responses by acting as an SA mimic. With respect to the ABA pathway, DF showed ambivalent effects with both positive and negative outcomes being found depending on the gene tested (Figures 7I and 7J). High concentrations of DSF, on the other hand, weakly but significantly suppressed ABA signaling. Although these results are consistent with a recent study showing that *Xcc* overcomes ABA-mediated stomatal immunity in Arabidopsis through a DSF-regulated virulence factor [67], they also seem to be at odds with the previously reported role of ABA in promoting BLB disease development [37]. However, given the tremendous increase in ABA-responsive gene expression in *Xoo*-infected rice leaves (up to 2,000-fold; Xu et al., 2013) and the rather weak effect of DSF on ABA signaling (less than 20% reduction relative to controls), one may question the biological significance of negative DSF-ABA interactions in determining the outcome of rice-*Xoo* interactions.

## Conclusions

**Figure 8:**
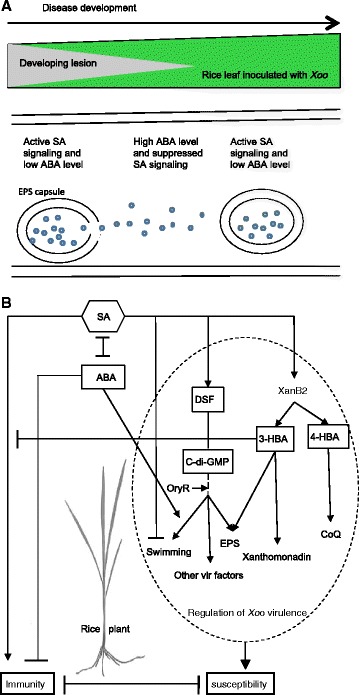
**Role and interplay of plant hormones and bacterial QS factors in molding rice-** ***Xoo*** **interactions. (A)** *Xoo* switches between sessile and motile growth stages by responding to and manipulating rice SA and ABA action. **(B)** Model illustrating how crosstalk between rice SA/ABA and the DF and DSF QS systems shapes *Xoo* infection biology and determines pathological outcomes. Sharp arrows represent stimulatory effects, blunt arrows depict antagonistic interactions.

Collectively, our data suggest a potential cross-communication between the DF and DSF *Xoo* QS circuits and the rice SA and ABA signaling pathways. In Figure 8, we propose a spatiotemporal model illustrating the role and interplay of plant hormones and bacterial QS factors in regulating rice-*Xoo* interactions. We speculate that upon leaf entry, *Xoo* is faced with high levels of SA in the xylem, which restricts its growth and motility and is deployed by rice as an efficient defense mechanism. *Xoo*, however, appears to perceive SA as a QS agonist with resultant activation of the DSF and DF QS systems, a process that at least partially depends on the LuxR solo protein OryR. SA-induced DF and DSF signaling in turn triggers production of EPS, xanthomonadin and CoQ, which collectively contribute to bacterial virulence. At this stage, *Xoo* are sessile and according to recent reports in the literature most likely growing within a biofilm [41,68]. Via a yet-to-be-defined mechanism, successful *Xoo* bacteria may then trick the plant into synthesizing ABA, which together with SA-induced DF contributes to suppression of SA signaling. As bacterial communities grow in size, cells that reside in the innermost layers of the biofilm may not have access to nutrients or may suffer from accumulation of toxic waste products. In either of these cases, or when environmental nutrients become limited, bacteria are likely to respond by returning to their planktonic mode of existence. With the aid of ABA and OryR and free from SA suppression, bacteria that are released from the biofilm swim to a further site, where they are again confronted with high SA levels. By repeating this process over and over again, *Xoo* bacteria may gradually spread along the rice xylem vessels, causing typical leaf blight symptoms as they progress. Although the specific mechanisms involved in various steps of the model remain to be elucidated, our data draw important inferences suggesting that *Xoo* responds to both a bacterial QS signaling system and a rice hormone signaling circuit to fine-tune virulence gene expression at different stages of infection. Moreover, besides reinforcing the earlier contention that QS is a bidirectional process influenced by both plant and microbial signals [65], our findings highlight the importance of plant hormones in modulating bacterial virulence and uncover interkingdom signaling as an important regulatory aspect of plant-bacteria interactions.

## Methods

### Plant growth conditions and chemical treatments

The rice line used in this study, Taipei, was a kind gift from Dr. He (Shanghai Institute of Biological Science, China). Plants were grown in commercial potting soil (Structural, Type 1) under greenhouse conditions (28 ± 4°C, 16/8 light regimen), and fertilized with 0.5% ammonium sulphate and 0.5% iron sulphate every week.

Both DF and DSF were purchased from Sigma (USA and Switzerland, respectively). DF was directly dissolved in distilled water, while DSF was first dissolved in a few drops of methanol and then diluted in distilled water. For chemical treatments, the two youngest leaves of 6-week-old plants (7- to 8-leaf stage) were detached, cut into 3-4 cm segments and floated overnight on sterile distilled water to eliminate the possible effects of wounding stress. The following day, leaf segments were floated on aqueous solutions containing various concentrations of DF or DSF and incubated for 8 or 24 hours at 28°C. For all experiments, leaf pieces from at least four individual plants were pooled and randomly distributed among treatments.

### Bacterial strains and growth conditions

The *Xanthomonas oryzae* pv. *oryzae* (*Xoo*) strains and plasmids used in this work are listed in Additional file 2. All strains were routinely grown on PSA plates (1% peptone, 1% sucrose, 1.5% bacto agar) at 28°C. When necessary, antibiotics were added at the following concentrations: ampicillin, 75 μg/ml; kanamycin, 50 μg/ml; gentamycin, 15 μg/ml; tetracycline, 10 μg/ml. For RNA isolation as well as EPS bioassays, single bacterial colonies grown on PSA plates were transferred to 30 ml of peptone yeast broth (PY; 0.8% peptone, 0.2% yeast extract, 0.5% glucose, 0.2% K_2_HPO_4_, 0.05% KH_2_PO_4_, 1 mM MgSO_4_) containing the appropriate antibiotics and shaken at 180 rpm for 24 h at 28°C. Precultures were then diluted (OD_595_ = 0.08) in 50 ml PYB containing antibiotics and hormones or corresponding solvents, shaken for 24 h at 180 rpm, and subjected to the various assays described below. For EPS and xanthomonadin assays performed on PSA-grown bacteria, all cultures were grown for 4 days at 28°C, after which cells were scraped off the plates and resuspended in 15 ml sterilized water.

### Swimming assay

*Xoo* precultures were grown as described above and diluted to a final density of 1 × 10^9^ CFU/ml. After carefully placing 3-μl suspension droplets in the centre of soft swimming plates (0.03% peptone, 0.03% yeast extract, 0.3% agar), plates were incubated at 25°C and evaluated after either 4 or 7 days by measuring the diameter of the swimming zone.

### Quantification of exopolysaccharides (EPS)

*Xoo* culture supernatant was collected by centrifuging at 12000 × *g* for 10 min. Afterwards, the supernatant was mixed with 1.0% potassium chloride and two volumes of absolute ethanol and incubated overnight at -20°C. After centrifuging, EPS pellets were dried for 24 h at 55°C and weighed. All values were expressed relative to the cell density.

### Xanthomonadin quantification

Xanthomonadins were quantified according to the method described by [24]. In brief, *Xoo* cells were collected from 4 ml suspension by centrifuging at 12,000 × *g* for 5 min and mixed with 1 ml absolute methanol. The mixtures were then incubated for 10 min in darkness in a rotating shaker and subsequently centrifuged at 12,000 × *g* for 5 min. The xanthomonadin pigments present in the supernatant were quantified by measuring OD_445_ and expressed relative to the cell density before the assay (OD_595_).

### Gene expression analysis

XKK12 cultures were grown as mentioned above and sampled when the OD_595_ reached 2.0. Total RNA of *Xoo* cells and rice leaves was isolated using TRIZOL (Sigma) and treated with Turbo DNase (Ambion) to remove genomic DNA contamination. First-strand cDNA was synthesized from total RNA using Multiscribe reverse transcriptase (Applied Biosystems) and random primers following the manufacturer’s instructions. Quantitative PCR amplifications were conducted in optical 96-well plates with the Mx3005P real-time PCR detection system (Stratagene), using Sybr Green master mix (Fermentas) to monitor dsDNA synthesis. The expression of each gene was assayed in duplicate in a total volume of 25 mL including a passive reference dye (ROX) according to the manufacturer’s instructions (Fermentas). For bacterial samples, the thermal profile used consisted of an initial denaturation step at 95°C for 5 min, followed by 45 cycles of 95°C for 10 sec, 60°C for 30 sec and 72°C for 30 sec. To verify amplification of one specific target cDNA, a melting-curve analysis was included according to the thermal profile suggested by the manufacturer (Stratagene). The amount of bacterial RNA in each sample was normalized using 16S rRNA (PXO_rna52) as internal control. For plant samples, *eEF1α* (Eukaryotic elongation factor 1alpha) was selected as reference gene [69] and we used the same thermal profile as described previously [37]. All data were analyzed using Stratagene’s Mx3005P software. Nucleotide sequences of all primers used are listed in Additional file 3.

### β-glucuronidase gene promoter activity assays

This assay was performed exactly as described by Ferluga and Venturi [28]. In brief, one ml XKK12 (pORY122) culture (OD_595_ = 2.0) was centrifuged to collect cells, which were subsequently resuspended in 600 μl GUS buffer (pH 7.0) and mixed with 23 μl 3% sodium lauryl sarcosinate and 23 μl 3% Triton X-100 in GUS buffer. Following incubation for 30 min at 37°C, 100 μl 25 mM p-nitrophenyl-β-D-glucuronic acid (PNPG) was added. The reaction was stopped by the addition of 280 μl 1 M NaCO_3_ after sufficient yellow color had developed. Both the optical densities at 595 nm of the *Xoo* cultures and the OD_415_ of the PNPG-treated samples were measured and one Miller unit of β-glucuronidase activity was defined as follows: one Miller unit = 1,000 × {[OD_415 PNPG_ – (1.75 × OD_595,PNPG_)]/(t × v × OD_595_)} where t is the time of the reaction (in minutes), v is the volume of cells used in the reaction mixture (in ml), OD_415 PNPG_ is the absorbance by PNPG measured after the reaction, OD_595,PNPG_ is a measure of the cell density after the β-glucuronidase reaction (used as a correction for light scattering by cell debris), OD_595_ is a measure of cell density just before the assay, and 1.75 is the corresponding correction factor. All measurements were performed in triplicate.

### Quantification of QS factors

XKK12 cultures were grown as mentioned above and sampled when the OD_595_ reached 2.0. DSF, BDSF, CDSF, DF and 4-HBA were all measured using HPLC exactly as described by [20,42].

## Additional files

Additional file 1:
**Concentration (pmol/g Fresh Weight) of SA in** ***Xoo*** **-inoculated rice leaves.**
Additional file 2:
**List of bacterial strains and plasmids used in this study.**
Additional file 3:
**Primer sequences used in this study.**

## Abbreviations

SA: Salicylic acid
QS: Quorum sensing
NaSA: Sodium salicylate
DSF: Diffusible signal factor
DF: Diffusible factor
ABA: Abscisic acid
BLB: Bacterial leaf blight
EPS: Exopolysaccharides
AIs: Autoinducers
Xoo: *Xanthomonas oryzae* pv. *oryzae*
Xcc: *Xanthomonas campestris* pv. *campestris*
HBA: Hydroxybenzoic acid
CoQ: Coenzyme Q
AHL: N-acyl homoserine lactone
PSA: Peptone sucrose agar
