# Supplementary material for: Phytohormone-mediated interkingdom signaling shapes the outcome of rice-Xanthomonas oryzae pv. oryzae interactions
Source: BMC Plant Biol. 2015 Jan 21;15:10. doi: 10.1186/s12870-014-0411-3 (PMC4307914; doi:10.1186/s12870-014-0411-3)
Supplement: Additional file 1: — Concentration (pmol/g Fresh Weight) of SA in Xoo -inoculated rice leaves. [file 12870_2014_411_MOESM1_ESM.pptx]

## Slide 1
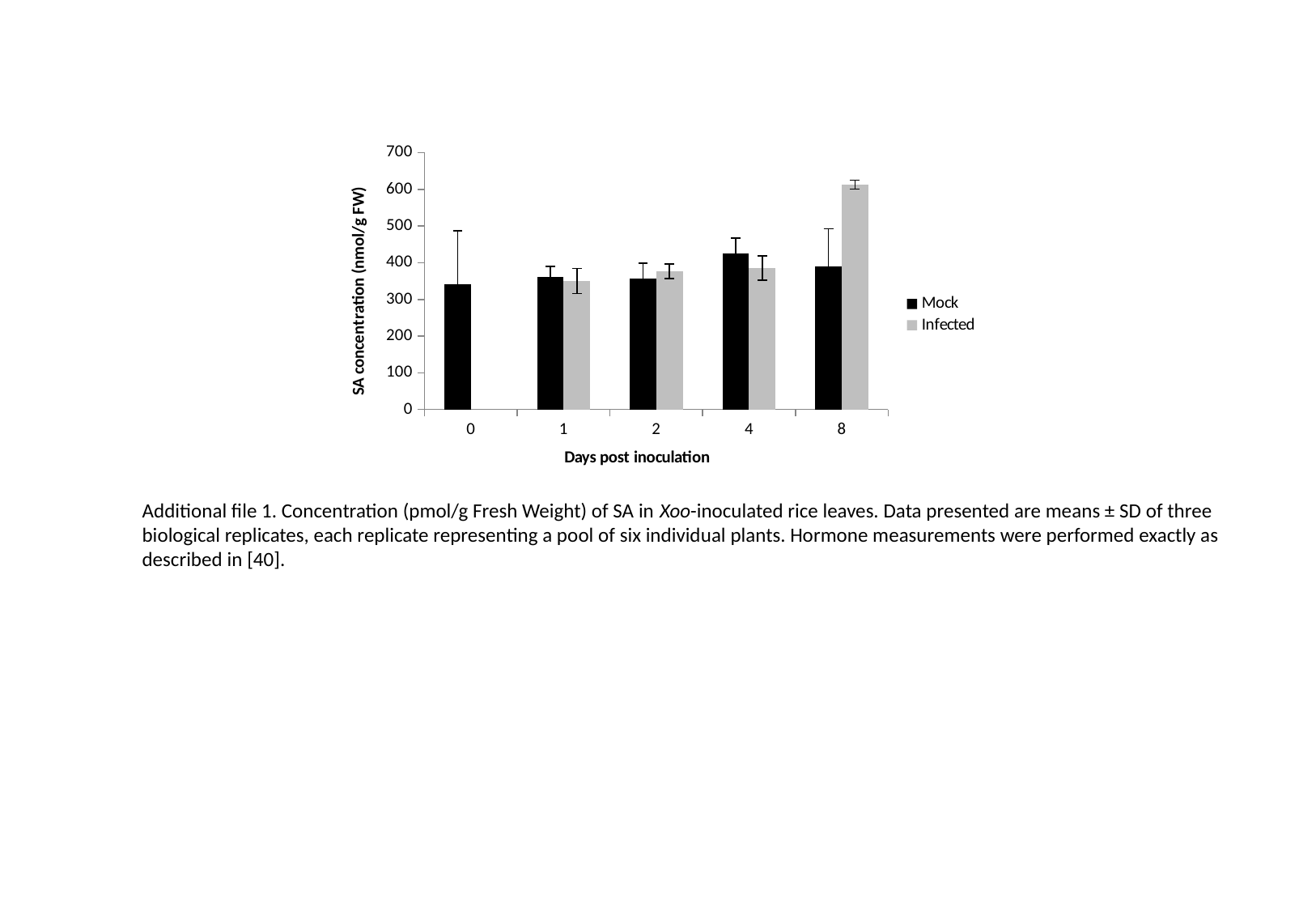

### Chart
| Category | Mock | Infected |
|---|---|---|
| 0.0 | 341.64 | None |
| 1.0 | 360.32 | 349.83 |
| 2.0 | 357.21 | 376.59 |
| 4.0 | 425.43 | 385.44 |
| 8.0 | 389.52 | 613.35 |Additional file 1. Concentration (pmol/g Fresh Weight) of SA in Xoo-inoculated rice leaves. Data presented are means ± SD of three biological replicates, each replicate representing a pool of six individual plants. Hormone measurements were performed exactly as described in [40].
